# The influence mechanism of industrial policies on Chinese companies' cross-border M&A decision-making

**DOI:** 10.1038/s41598-023-43445-8

**Published:** 2023-09-27

**Authors:** Yalan Shen, Mengqi Yang, Hongyu An, Kailun Li

**Affiliations:** 1 University of International Business and Economics, Beijing, 100029 China; 2 School of Finance, Anhui University of Finance and Economics, Bengbu, 233030 China; 3 School of Journalism and Communication, Shanghai International Studies University, Shanghai, 200083 China; 4 School of Management Engineering and Business, Hebei University of Engineering, Handan, 056009 China

**Keywords:** Environmental social sciences, Environmental economics

## Abstract

This study explored the potential links between Chinese industrial policy and cross-border mergers and acquisitions by Chinese firms from 2009 to 2019. Based on describing China's industrial strategy and evaluating the then-current situation of Chinese enterprises' cross-border mergers and acquisitions, this empirical study constructed a two-way fixed-effect panel model and an intermediate effect model to assess the mechanism of industrial policy's influence on Chinese enterprises' cross-border mergers and acquisitions decisions. The findings were as follows: (1) Industrial policy could promote the implementation of cross-border mergers and acquisitions of Chinese enterprises; (2) By easing financial restrictions, industrial policy could improve firms' access to capital and encourage cross-border mergers and acquisitions. (3) Industrial policies could promote the high political relevance of state-owned enterprises, thus promoting the success of transnational mergers and acquisitions of enterprises. Therefore, it was significant to promote the transformation of industrial policy from subsidy-oriented to performance-oriented and rationally evaluate the risks and benefits of M&A for enterprises to complete cross-border M&A.

## Introduction

In recent years, with the development of the global economy, and the rise of anti-globalization and trade protectionism, Chinese enterprises faced many uncertain factors in cross-border mergers and acquisitions, and the difficulty of cross-border mergers and acquisitions also constantly increased. On the other hand, cross-border mergers and acquisitions could help companies acquire the core technologies and management systems they needed, thereby narrowing the gap with developed countries more quickly. So, as the difficulty of cross-border mergers and acquisitions continued to increase, its significance became increasingly apparent. How to better protect enterprises from cross-border mergers and acquisitions and thereby improve the success rate of mergers and acquisitions had become increasingly important.

Given the risen number of publicly traded companies that had engaged in mergers, acquisitions, and restructuring operations, the role of industrial policy in China's capital market was critical. An industrial policy of a country was its official strategic effort to encourage the development and growth of all or part of the economy, often focusing on all or part of the manufacturing sector. The government took measures aimed at improving the competitiveness and capabilities of domestic firms and promoting structural transformation. Industrial policy referred to the decision on whether to control the management of listed companies based on industrial assistance policies. Chinese companies that merged and acquired cross-border were essential to the execution of their globalization strategy and were related to China's open economic construction as well as its status and influence on the global stage^1,2^. Even though China's industry had experienced rapid economic expansion driven by industrialization and urbanization, its economy was expected to continue to grow at a reasonable rate. Consequently, an increased number of listed companies had engaged in mergers, acquisitions, and restructuring activities, further emphasizing the importance of industrial policy in China's capital market.

After the global financial crisis, many foreign companies had faced operational difficulties or even bankruptcy and had sought financial support from overseas companies, creating an opportunity for Chinese companies to acquire assets at lower prices and expand their international market presence. According to data on transnational M&A by Chinese companies provided by the Ministry of Commerce, Chinese companies had completed a total of US$38.6 billion in cross-border M&A in 2008, followed by US$21.6 billion in 2009, and US$30.1 billion in 2010. By 2018, Chinese companies had completed US$70.26 billion worth of international mergers and acquisitions, accounting for 54.12% of total outbound investment. In addition, the China Securities Regulatory Commission had reported that in 2017, the Mergers and Reorganization Review Committee of the China Securities Regulatory Commission had reviewed a total of 176 restructuring transactions involving Chinese companies, including 7 restructuring and listing transactions, 167 single share purchase asset purchase transactions, and 2 single absorption merger transactions, involving a total of 169 companies. In 2019, the Commission had reviewed a total of 148 restructuring transactions and had approved 75, and in 2020, the Commission had reviewed 127 transactions and had approved 54.

Several scholars argued that mergers and acquisitions could optimize asset structure and increase the value of the firm. However, the performance of companies listed on the Chinese stock exchange after mergers and acquisitions was not always consistent with this theory^3–7^. Given China's unique system background, the motivations of companies for mergers and acquisitions were complex and the factors influencing merger decisions were diverse^8^. The company's profit growth targets, regulatory policy arbitrage, and administrative intervention by local governments could all affect merger and acquisition decisions^8,9^. Therefore, it was crucial to maximize corporate mergers and acquisitions and use them to become bigger and stronger, especially in the context of the Chinese system. To achieve this, it was important to study the factors that influenced merger and acquisition decisions, examine whether premiums followed mergers and acquisitions, and analyze the performance of companies after mergers and acquisitions. Particularly for large state-owned enterprises and enterprise groups like Sinochem, cross-border corporate M&As were drawing greater attention. Especially in the expansion of Chinese transnational corporate M&A. This, as Pan H. et al. said, was because M&A in transnational was a traditional way of enterprise development and growth, and a result of industrial policy to support the development of capital markets^2^.

The book "Japanese Industrial Policy", edited by Ryuichiro Komiya, a Japanese economist in the 1980s, provided the earliest and most comprehensive analysis of industrial policy in Japan^10,11^. According to Komiya, the term "industrial policy" had not been used in Japan until the 1970s. In the late 1940s to early 1950s, "industrial rationalization" had been the preferred term, while in the 1970s, especially after the oil crisis, "industrial structural adjustment" became widely used. Around 1975, Japanese scholars had started to use the phrase "industrial policy" to refer to the industrial development policies from the 1950s to the 1970s. The implementation of industrial policy in Japan had involved various measures. Policy banks had received significant lending support and direction from institutions like the Japan Development Bank and the Japan Export–Import Bank. Other measures had included accelerated depreciation and tax incentives to encourage rapid equipment upgrading, foreign exchange preference to encourage technology imports, and tariffs and trade quotas to control tariff trade. However, the strategic and selective nature of industrial policy meant that support for certain industries and enterprises often directly or indirectly suppressed the growth of other industries and enterprises. The advocates of the new growth theory and the new trade theory, including Aghion, Roderick, and Helpmann, had classified industrial policies into different types, such as horizontal and vertical, functional and selective. China's policy system at that time had a standard formulation: "Macro policy was stable, industrial policy was precise, the micro policy was active, reform policy was practical, and social policy was bottom-supporting." Compared with the scope of industrial policy mentioned above, China's "quasi" industrial policy had been separated from "active" micro policy, which indicated that China's industrial policy mainly referred to selection.

The interplay among economic sectors, the global organization of production, and the characteristics of technical systems that had substantial consequences for industrial policy were the three main characteristics that defined the modern global economy. The interaction of these features not only created novel accumulations, but also allowed for new value creation and the reconstruction of value capture models and dynamics. New regimes of accumulation and modifications in the dynamics of value creation as well as capture had resulted from the restructuring of the system of global production^10^. The phenomenon of financialization had led to a spiral of underinvestment that threatened the sustainability of capitalist economies. In response to these challenges, countries needed to adopt policies to enhance their competitiveness under the new global political regime. However, the interdependencies between these three global transformations might have had unforeseen consequences that required careful attention. The emergence of new changes in the global economy had resulted in the creation of a new industrial policy theory, which focused primarily on a set of policies aimed at promoting innovation, fostering competitive development, enhancing cohesion, and improving innovative capacity in an open economy. These policies were implemented by a variety of institutional actors. The government utilized industrial policy to affect the industrialization process and direct the restructuring mechanism, which was considered in this new framework as the core of the transformation of the whole economy. The new approach to industrial policy placed greater emphasis on knowledge and innovation and recognized the critical role of innovation policy in promoting sustainable development and enhancing a country's global competitiveness^12–14^.

The modern industrial economy was characterized by the irreversibility of production commitments^15^, and firms might have resorted to predatory pricing or merger and acquisition strategies to mitigate environmental uncertainty^16,17^. The three main economic policies, namely industrial, fiscal, and monetary policies, were considered important tools of government macroeconomic regulation that worked together to adjust the economy^18^. Policymakers could reduce uncertainty and encourage investment commitment and innovation by developing industrial policies that used demand stabilization tools. To ease financial constraints and promote cross-border M&A activities, enterprises could use various industrial policy tools, such as improving bank credit and capital market equity financing methods, to obtain funds more easily or at a lower cost, and reduce the cost of merger and acquisition financing. Fiscal and tax incentives and tax breaks could also provide more resources, reduce merger integration and transaction costs, and ultimately reduce the opportunity cost of mergers and acquisitions^19^. The financial constraints faced by companies supported by the national industrial strategy could be eased with the support of a development-friendly Chinese industrial policy set by the government. Companies were increasingly inclined to engage in transnational mergers and acquisitions in order to seek advanced technology and achieve technological upgrading. Therefore, industrial policies could encourage companies to conduct mergers and acquisitions in the cross-border sector by reducing the then-current restrictions on financing. We expected that industrial policies would influence the company's M&A decisions in a cross-country context and its performance in overseas markets.

Past research on industrial policy mainly focused on whether industrial policy could promote green growth, whether it could promote strategic emerging industries, and its relationship with other policies. Specifically, Nilsson et al. mainly studied the impact of industrial policies on green growth, and different scholars had different views on the two^20^. Chick introduced a general four-stage model describing the industrial policy process in China^21^. Weiss put the policy trajectory of China's industrial policy, technological innovation, and upgrading in the context of institutional economic analysis and analyzed the relationship between China's industrial policy and strategic emerging industries^22^. Bailey considered the appropriate spatial scale of industrial policy, questioning whether the policy should have been concentrated in a specific place for spatially concentrated enterprise clusters^23^. For the study of multinational enterprises and industrial policies, Feng had studied whether the potential value of investment had been worth adopting active industrial policies^6^. Wang et al. had found that subsidiary policy was not only the product of adapting to the domestic and host country institutional environment but had also been shaped by the multiple power relations of multinational subsidiaries^24^. Zhou had found that compensation of R&D for their domestic base might have encouraged industrial policy reform in their own countries^25^. Xu et al. had linked industrial policy and supplier learning capability through surveys of multinational enterprises. Industrial policy had helped establish a local component supply base and had facilitated market access for assemblers^26^.

The gaps between this study and the previous ones were as follows: in the prior research, most scholars had focused on developing industrial policy in a certain country, the comparison with other policies, and whether industrial policy could promote economic growth. The research on transnational enterprises and industrial policy primarily concentrated on whether the use of industrial policy could encourage the development of enterprises and whether the transformation of industrial policy could be realized. Studies on the relationship between industrial policy and the cross-border M&A premium of Chinese enterprises were present. This paper had analyzed the relationship between industrial policy and the cross-border M&A premium of Chinese enterprises and its theoretical mechanism. It had examined whether there was a correlation between the success rate of cross-border M&A of Chinese enterprises and industrial policy. Targeted adjustment of cross-border M&A policies and related systems had promoted the construction of a mature cross-border M&A market.

The research contribution of this paper had been that, at that time, academic circles mostly focused on investment, financing, innovation, and other aspects of the impact of industrial policies on enterprises' micro-behaviors while paying little attention to the relationship between industrial policies and cross-border mergers and acquisitions of enterprises. Previous studies had mostly concentrated on the role path of industrial policies in cross-border mergers and acquisitions of enterprises. Therefore, this project had taken industrial policies as the entry point. An in-depth study of the mechanism of industrial policy on the performance of cross-border mergers and acquisitions had helped to improve the relevant theories. In addition, based on a literature review and theory, this paper had analyzed and summarized the theoretical mechanism of enterprises' cross-border mergers and acquisitions driven by industrial policies. Industrial policies had affected cross-border mergers and acquisitions through financial constraints and policy-related mechanisms. By relaxing financial restrictions, industrial policies had improved companies' ability to obtain funds and had encouraged cross-border mergers and acquisitions. It had been helpful to further understand the relationship between industrial policy and transnational mergers and acquisitions.

## Theoretical basis analysis and research hypothesis

### The direct impact of industrial policy on cross-border mergers and acquisitions

In recent years, the direct impact of industrial policy on cross-border mergers and acquisitions had been extensively studied^27–31^. Some researchers had concluded that industrial policies could directly promote listed cross-border M&A, while others believed that industrial policies hindered listed companies' cross-border M&A. The specific corresponding research process was as follows:

Haffert had pointed out that when a nation crafted explicit industrial policies for a specific sector, signaling strong support and encouragement, it was often accompanied by a series of favorable measures, such as tax reductions, preferential loans, and R&D funding assistance^27^. These actions aimed to enhance the core competitiveness of industry enterprises and attract external investments, particularly in the realm of cross-border mergers and acquisitions (M&A). Kenderdine had found through research that cross-border M&A was widely regarded as an efficient mechanism for technology and knowledge transfer^28^. A primary objective of industrial policies might have been the introduction of cutting-edge technology and managerial expertise to foster rapid domestic industrial growth. Under this strategic impetus, cross-border M&A offered foreign enterprises new market entry opportunities while also channeling pivotal technology and professional knowledge to the host country. Wen & Zhao suggested that industrial policies might have been designed to tap into new market potentials or to amplify the competitiveness of existing markets, thus acting as magnets for foreign direct investments^29^. In that context, cross-border M&A emerged as an especially appealing strategy, allowing foreign firms to seamlessly integrate into new markets, capitalize on policy dividends, and swiftly establish market dominance. Zhou emphasized that clear industrial policies provided businesses with a lucid developmental direction, vividly showcasing the strategic vision of the government. For foreign enterprises, this clarity effectively mitigated uncertainties inherent in investments, rendering M&A decisions more precise and efficient^30^. Chen et al. noted that in the face of policies that promoted the growth of a specific sector, foreign enterprises often opted for cross-border M&A as a means to merge with local entities, aiming for synergy^32^. Such integrative strategies not only fortified a firm's market position but also potentially enhanced its production efficiency, innovation capacity, and market responsiveness. Yülek contended that industrial policies could be likened to a credit endorsement bestowed by the government upon enterprises, offering external investors a clear economic trajectory. Such assurances significantly diminished investor risk apprehensions, thereby drawing increased capital participation^33^. Barbieri et al. (2021) argued that, under the guidance and encouragement of industrial policies, corporate actions became more aligned with national strategic objectives^34^. This not only bolstered corporate legitimacy and credibility but might also have further ignited their motivation for international expansion. With governmental backing, these companies might have been more inclined to pay a premium in cross-border M&A to achieve broader policy or business goals. Zhang et al. emphasized that corporate investment strategies were frequently influenced by peers in the same industry^31^. When certain firms proactively responded to industrial policies and embarked on cross-border M&A initiatives, a ripple effect or "peer effect" might have ensued across the industry. Astute industrial policies aimed to eradicate barriers to cross-border investments, reshaping the macro-business environment through institutional incentives, thereby reducing uncertainties and bolstering firms' overseas investment aspirations and capabilities.

However, while some key industries or industries with crucial technologies received support from industrial policies, these policies might have hindered cross-border mergers and acquisitions (M&A) due to national security considerations^35^. To protect domestic industries from external competitive pressures and ensure the global competitive position of domestic enterprises, governments might have employed industrial policies to deter acquisitions by foreign companies^36^. Furthermore, to prevent the outflow of critical technologies, governments restricted foreign acquisitions in certain high-tech sectors^37^. Additionally, some countries had rigorous review and approval processes for cross-border M&A, potentially involving multiple governmental departments and taking considerable time to finalize, which resulted in delays or even failures of M&A transactions^38^.

Therefore, on one hand, industrial policies, by offering tax and loan incentives, facilitated cross-border mergers and acquisitions (M&A) and technology transfer, attracting foreign investment to enter new markets and bring in technical expertise. Additionally, these policies reduced investment uncertainties, thus potentially bolstering the willingness of companies to engage in cross-border M&A. On the other hand, due to national security, technological protection, prevention of technology outflow, and the maintenance of competitive positioning, the review and approval processes in some countries might have delayed or even prevented M&A. Consequently, industrial policies might also have inhibited cross-border M&A. Based on the aforementioned theoretical analysis, this study put forth the following hypotheses.

#### H1.1

Industrial policies could promote cross-border merger and acquisition decisions of listed companies.

#### H1.2

Industrial policy hindered the cross-border M&A decisions of listed companies.

### The indirect effects of industrial policy on cross-border mergers and acquisitions: financial constraint mechanism and political relevance

This study primarily investigated how industrial policies facilitated cross-border mergers and acquisitions by alleviating financial constraints and enhancing political relevance. The specific analyses were as follows:

Industrial policies exerted a profound influence on enterprises, especially evident in their financing activities. These policies were geared towards alleviating financing constraints, thereby bolstering corporate cash flows. With ample funds, businesses could make decisions more promptly, capitalizing on merger and acquisition opportunities without undue concern for cash shortages^39^. However, businesses often confronted the challenge of incomplete external information when making investment decisions. The execution of industrial policies afforded companies a more stable and defined investment environment. Moreover, when companies had access to a larger pool of funds and financing channels, they were better poised to counter potential risks stemming from information gaps, making them more predisposed to seek merger opportunities^40^. To champion specific industries and enterprises, governments frequently incorporated credit intervention measures into their industrial strategies. Such interventions, like interest rate cuts, fiscal subsidies, or preferential loans, were intended to decrease firms' financing costs, thus making it more feasible for them to procure funds. Consequently, businesses with substantial capital reserves were at an advantage in M&A negotiations, allowing them to pitch more competitive offers and thereby securing a commanding position in the M&A landscape^41^. Furthermore, local governments, guided by industrial policies, often encouraged banks to extend loans to particular industries or businesses. This collaboration further fostered the fusion of bank credit with industrial capital, significantly easing the loan procurement hurdles for specific sectors. This not only mitigated financing constraints for companies but also facilitated their international M&A concerning technology and specialized knowledge, leading to swift knowledge and technology transfer and diffusion^42^. Industrial policies assisted companies on various fronts, especially those in their growth phase, in navigating their financing dilemmas. Moreover, when the leadership of a company harbored a strong inclination for investment and expansion, such policy backing intensified their investment decision-making vigor. Firms with a high appetite for risk were better equipped to navigate cultural and market shifts during mergers, further propelling the evolution of international M&A^43^.

Industrial policies provided state-owned enterprises (SOEs) with a set of conditions favorable for cross-border mergers and acquisitions (M&As). Firstly, the preferential financing conditions endowed SOEs with financial flexibility in cross-border M&As. This financial support not only reduced capital costs but also equipped SOEs with strong bargaining power during M&A negotiations. Additionally, they could afford top-tier financial advisors and legal teams for comprehensive due diligence, thus enhancing the likelihood of successful acquisitions^44^. Simultaneously, industrial policies highlighted sectors or industries deemed as national strategic development priorities. Owing to the close political affiliation between SOEs and the government, these enterprises frequently garnered robust government support in cross-border M&As. Such governmental intervention often manifested as diplomatic interactions with the target nation, efficiently mitigating potential political and cultural impediments during the acquisition process and offering SOEs a clear direction for their international M&A initiatives^44^. The government also furnished SOEs with profound insights into the economic and legal landscapes of the target countries. This aided them in circumventing potential risks in acquisitions, ensuring their activities were lawful and compliant. Moreover, SOEs might have benefited from specialized government backing and protection, further alleviating M&A risks^45^. The dominant market position of SOEs in their domestic markets yielded abundant cash flows and formidable bargaining power. Concurrently, this elevated their international brand recognition and reputation. This not only amplified their bargaining strength in cross-border M&As but also made them more appealing to potential acquisition targets^46^. On the technological front, industrial policies encouraged SOEs lagging in critical technological sectors to leverage cross-border M&As as a means to acquire advanced technologies and expertise, accelerating technological advancements and innovation. Through this approach, SOEs could better adapt to and penetrate new markets, enhancing their efficiency in international operations^47^. Lastly, industrial policies advocated the establishment of bilateral or multilateral cooperation frameworks. This not only streamlined the approval and execution process for cross-border M&As but also offered SOEs an effective platform to access information on potential acquisition targets. Such clear guidance and backing significantly reduced obstacles in M&As, increasing the chances of successful acquisitions.

In summation, industrial policies, by availing technical R&D and financial support to businesses, safeguard their long-term investment activities and wholesome growth. As companies find it easier to garner funds, their investment and M&A potential invariably amplifies. In addition, industrial policy promoted the cross-border M&A of SOEs through preferential financing, diplomatic support and professional consulting. Based on the aforementioned theoretical analysis, this study puts forth the following hypotheses.

#### H2.1

Industrial policies can alleviate financing constraints, thereby promoting cross-border merger and acquisition decisions of listed companies.

#### H2.2

Industrial policies can enhance industry political relevance, thereby promoting cross-border merger and acquisition decisions of listed companies.

## Standards and processes

### Benchmark design

This section presented the theoretical framework for examining industrial policy's impact on companies' international merger and acquisition decisions. Additionally, it offered an empirical examination of how industrial policy and these company choices interacted. The use of panel data allowed the analysis to be conducted on a large sample size; the use of these data met all the ethical norms of academic research and helped to increase the reliability of estimates and tests. By taking into consideration the unique distinctions or "heterogeneity" produced by this problem, panel data also aided in reducing the issue of missing variables with no temporal variation. In order to correctly measure how industrial policy affected M&A choices of cross-border, this study used a panel data technique.

The baseline regression model specified in this article was:

1 $$\mathop {{\text{cbma}}}\nolimits_{{{\text{it}}}} { = }\mathop {\text{a}}\nolimits_{{0}} { + }\mathop {\text{a}}\nolimits_{{1}} \mathop {{\text{policy}}}\nolimits_{{{\text{it}}}} { + }\mathop {\text{a}}\nolimits_{{2}} \mathop {\text{X}}\nolimits_{{{\text{it}}}} { + }\mathop {\text{m}}\nolimits_{{\text{i}}} { + }\mathop {\text{g}}\nolimits_{{\text{t}}} { + }\mathop {\upvarepsilon }\nolimits_{{{\text{it}}}}$$

The subscript t stood for time and the subscript i for company. If a company had successfully executed a cross-border acquisition, the dependent variable, cbma, was set to 1; otherwise, it was set to 0. A binary variable called "policy" was 1 if the industry of the listed company was favored through industrial policy and 0 otherwise. X was the control variable of transformation of the low carbon economy. Symbol μ and symbol γ symbolized the individual effects and the effects fixed in time, respectively, while ε symbolized the time-varying random error terms. The focus of this research was on the coefficient of the explanatory variable policy, which could be positive or negative. A significantly positive coefficient would indicate that industrial policy played an important role in promoting international mergers and acquisitions.

In this study, dynamic panel regression was used for robustness testing. Panel data allowed the simulation of the dynamic behavior of an individual. However, the current behavior of the individual was often determined by past behavior due to the presence of inertia. As a result, the panel model took the dependent variable's lagged value into account. To account for the possible dynamic adjustment and inertia of the company's merger and acquisition premium, this chapter considered a dynamic panel model for robustness testing:

2 $$\mathop {{\text{cbma}}}\nolimits_{{{\text{it}}}} { = }\mathop {\upalpha }\nolimits_{{0}} { + }\mathop {{\text{pccbmapre}}}\nolimits_{{\text{it - 1}}} { + }\mathop {\upalpha }\nolimits_{{1}} \mathop {{\text{policy}}}\nolimits_{{{\text{it}}}} { + }\mathop {\upalpha }\nolimits_{{2}} \mathop {\text{X}}\nolimits_{{{\text{it}}}} { + }\mathop {\upmu }\nolimits_{{\text{i}}} { + }\mathop {\upgamma }\nolimits_{{\text{t}}} { + }\mathop {\upvarepsilon }\nolimits_{{{\text{it}}}}$$

The above formula represented the dynamic panel model, where cbma represented Cross border acquisition, subscript t represented time, and subscript I represented the company. If a company successfully executed a cross-border acquisition, the dependent variable cbma was set to 1; otherwise, it was set to 0. X was the control variable for the transition to a low-carbon economy. The symbols μ and γ represented individual and time-invariant effects, respectively, and ε represented a random error term that varied over time.

To obtain consistent estimates, within-group estimation was insufficient due to the introduction of a lag of the explained variable. Therefore, several types of generalized method of moments (GMM) estimators were commonly used, including difference GMM, horizontal GMM, and system GMM. The difference GMM approach typically involved taking the first-order difference of the model to eliminate individual effects and then using an instrumental variable that was one period behind the explanatory variable's difference term. The variance term of the explanatory variable, usually delayed by a single period, served as the instrumental variable, and the GMM estimation was performed. In contrast, the horizontal GMM approach did not take the first-order difference and used the one-period difference term of the multi-period lagged explanatory variable directly as the instrumental variable for the GMM estimation. The system GMM approach combined the difference and horizontal equations into one system of equations for GMM estimation, which could increase estimation efficiency and estimate the coefficients of variables that did not vary over time.

### Inspection model of impact mechanism

The subscript t stood for time and the subscript i for company. The dependent variable in this part of the study, cbma, was a binary variable which adopted the value of 1 if the company had accomplished a cross-border acquisition and 0 otherwise. A binary variable called "policy" had a value of 1 if industrial policy supported the listed company's industry and 0 otherwise. X was the low-carbon economic transformation control variable. Symbol μ and symbol γ symbolized individual effects and time-fixed effects, respectively, while symbol ε symbolized the time-varying random error terms. The focus of this research was on the coefficient of the explanatory variable policy, which could be positive or negative. A significantly positive coefficient would indicate that industrial policy played an important role in promoting international mergers and acquisitions.

3 $$\mathop {{\text{cbma}}}\nolimits_{{{\text{it}}}} { = }\mathop {\upalpha }\nolimits_{{0}} { + }\mathop {\upalpha }\nolimits_{{1}} \mathop {{\text{policy}}}\nolimits_{{{\text{it}}}} { + }\mathop {\upalpha }\nolimits_{{2}} \mathop {\text{X}}\nolimits_{{{\text{it}}}} { + }\mathop {\upmu }\nolimits_{{\text{i}}} { + }\mathop {\upgamma }\nolimits_{{\text{t}}} { + }\mathop {\upvarepsilon }\nolimits_{{{\text{it}}}}$$

4 $${\text{long\_}}\mathop {{\text{asset}}}\nolimits_{{{\text{it}}}} { = }\mathop {\upbeta }\nolimits_{{0}} { + }\mathop {\upbeta }\nolimits_{{1}} \mathop {{\text{policy}}}\nolimits_{{{\text{it}}}} { + }\mathop {\upbeta }\nolimits_{{2}} \mathop {\text{X}}\nolimits_{{{\text{it}}}} { + }\mathop {\upmu }\nolimits_{{\text{i}}} { + }\mathop {\upgamma }\nolimits_{{\text{t}}} { + }\mathop {\upvarepsilon }\nolimits_{{{\text{it}}}}$$

5 $$\mathop {{\text{cbma}}}\nolimits_{{{\text{it}}}} { = }\mathop {\uplambda }\nolimits_{{0}} { + }\mathop {\uplambda }\nolimits_{{1}} \mathop {{\text{policy}}}\nolimits_{{{\text{it}}}} { + }\mathop {\uplambda }\nolimits_{{2}} {\text{long\_}}\mathop {{\text{asset}}}\nolimits_{{{\text{it}}}} { + }\mathop {\uplambda }\nolimits_{{3}} \mathop {\text{X}}\nolimits_{{{\text{it}}}} { + }\mathop {\upmu }\nolimits_{{\text{i}}} { + }\mathop {\upgamma }\nolimits_{{\text{t}}} { + }\mathop {\upvarepsilon }\nolimits_{{{\text{it}}}}$$

The explanatory variable, long asset, was included in Eq. (4) to measure the financing capability of each firm, and in this study, it was added based on the type (1). The stepwise regression approach was used to conduct the intermediation effect test, and the overall procedure was as follows: (1) If α1 was not significant, the intermediate effect test was terminated, indicating a weak causal relation of M&A activities in the transnational field and industrial policies of Chinese. The intermediate effects test was discontinued if β1 was not significant, which showed a weak causal link between industrial policy and funding for company constraints. To ascertain how industrial policy affected a company's ability to obtain funding, the regression equation of structure (4) was continued only if α1 was significant.

Subsequently, this paper constructed the influence mechanism model of political relevance. The model posited that industrial policy was likely to encourage companies with high political relevance to engage in cross-border reporting. The political relevance of a company was measured by its characteristics, which were used as proxies for a political relevance index. The specific parameters of the model were outlined below:

6 $$\mathop {{\text{cbma}}}\nolimits_{{{\text{it}}}} { = }\mathop {\upxi }\nolimits_{{0}} { + }\mathop {\upxi }\nolimits_{{1}} \mathop {{\text{policy}}}\nolimits_{{{\text{it}}}} { + }\mathop {\upxi }\nolimits_{{2}} \mathop {{\text{soe}}}\nolimits_{{{\text{it}}}} { + }\mathop {\upxi }\nolimits_{{3}} \mathop {{\text{policy}}}\nolimits_{{{\text{it}}}} {*}\mathop {{\text{soe}}}\nolimits_{{{\text{it}}}} { + }\mathop {\upxi }\nolimits_{{4}} \mathop {\text{X}}\nolimits_{{{\text{it}}}} { + }\mathop {\upmu }\nolimits_{{\text{i}}} { + }\mathop {\upgamma }\nolimits_{{\text{t}}} { + }\mathop {\upvarepsilon }\nolimits_{{{\text{it}}}}$$

In Eq. (6), the variable soe was included in Eq. (1) to represent policy relevance and was a dummy variable that had two possible values: 0 for state-owned enterprises and 1 for private companies. The coefficient on policy-soe was the main focus of this equation, which measured the interaction effect between industrial policy and soes. If this coefficient was statistically significant and positive, it indicated that industrial policy had a greater potential to influence cross-border M&A decisions through its impact on political relevance. In other words, soes were more inclined to use industrial policy to guide their decisions to merge or acquire across borders.

In order to test the robustness of the fixed effects model in this paper, the linear probability model (LPM) model was introduced. The model form was as follows:

7 $${\text{P(}}\mathop {{\text{cbma}}}\nolimits_{{\text{i}}} { = 1) = }\mathop \infty \nolimits_{i} { + }\mathop {\upomega }\nolimits_{{\text{i}}} \mathop {{\text{policy}}}\nolimits_{{\text{i}}} { + }\mathop {\upeta }\nolimits_{{\text{i}}} \mathop {\text{X}}\nolimits_{{\text{i}}} { + }\mathop {\upmu }\nolimits_{{\text{i}}}$$

In the above formula, cbma represented merger and acquisition decision, and the value of cross-border merger and acquisition conducted by listed companies within the year was 1, and the value of vice versa was 0. policy represented industrial policy, and if industrial assistance policy dominated the operation of listed companies, the value was 1, and the value of vice versa was 0. X represented the control variable; ω and η were the coefficient estimate vectors of the model; μ represented the random error term.

### Variable definition

This paper focused on the potential relationship between China's industrial policy and Chinese enterprises' cross-border mergers and acquisitions. The data of listed companies from 2009 to 2019 were selected, and the study of Chen was referenced. Cross-border mergers and acquisitions included Hong Kong, Macao, and Taiwan. The dependent variable was the merger decision, and the independent variable was industrial policy^21^. Explanatory Variables included Industrial Policy, Financial Constraints, and Political Relevance. To ensure the accuracy of the estimation, we used the following variables as control variables: firm size, return on assets, financial leverage, asset turnover, board size, CEO duality, main business revenue, revenue growth, executive compensation ratio, firm age, and retirement age.

#### Dependent variable

The M&A decision variable was the dependent variable of analysis in this study. If a company that was listed made a transborder merger or acquisition during the year, it became 1, otherwise it became 0. This variable was denoted as cbma.

#### Independent variables

Industrial Policy: the description content in the database divided industrial policy into two categories: encouragement and inhibition and gave a value of 1 to the industries that clearly supported development and encouraged development in the plan, and 0 to other industries. At the same time, based on the industry code of the Guidance on Industry Classification of Listed Companies (2012 edition), this paper matched the industrial policy data with the data of listed companies that engaged in mergers and acquisitions.

Financial Constraints: The financing capacity of enterprises was used to represent the financing constraints of companies. In this study, the long-term borrowing capacity and the ratio of business assets were used to denote financial constraints, and it was denoted as long asset.

Political Relevance: This study used a company's ownership structure as a proxy for its political relevance. If a company was state-owned, the enterprises in which the sum of state-owned capital and collective capital accounted for equal to or more than 50% of the total capital input were defined as state-owned enterprises, and the rest were defined as non-state-owned enterprises^48^. Then the binary variable with the soe symbol took the value 1; otherwise, it took the value 0.

Control Variables: firm size, return on assets, financial leverage, asset turnover, board size, CEO duality, main business revenue, revenue growth, executive compensation ratio, firm age, and retirement age. Table 1 presented the definition of each variable used in the analysis.

**Table 1:** Variable description.

	Variable representation	The calculation method	Symbolic meaning	The source of the data
Industrial policy	policy	$${\text{policy}} = \left\{ {\begin{array}{*{20}c} 1 \\ 0 \\ \end{array} } \right.$$	A binary variable that is either 1 or 0 depending on whether an industrial assistance policy governs the listed company's operations	Compiled in accordance with the work report of the government
Cross-border acquisition of virtual variables	cbma	$${\text{cbma = }}\left\{ {\begin{array}{*{20}c} {1} \\ {0} \\ \end{array} } \right.$$	If a company that is listed has made a transborder merger or acquisition during the year, it becomes 1, otherwise it becomes 0	SDC platinum database
The size of the listed company	size	size = ln TAC	TCA represents the total assets of the company	Cathay Pacific database and author calculations
Returns on listed companies' net assets	roe	roe = NI/TAC	NI stands for enterprise net income; TCA represents the total assets of the company	Cathay Pacific database and author calculations
Financial leverage of listed companies	asset debt	asset debt = TL/TAC	TL represents the total liabilities; TCA represents the total assets of the company	Cathay Pacific database and author calculations
Return on assets of listed companies	roa	roa = NP/TAC	NP represents the net profit; TCA represents the total assets of the company	Cathay Pacific database and author calculations
The size of a listed company's board of directors	board dimensions	board dimensions = BD	BD represents the number of directors of companies listed on the stock exchange	Cathay Pacific database
Listed companies are one of two jobs	dual	$${\text{dual = }}\left\{ {\begin{array}{*{20}c} {1} \\ {0} \\ \end{array} } \right.$$	If it is a listed company, take 1, and if it is an unlisted company, take 0	Cathay Pacific database and author calculations
The principal revenue of a publicly traded company	revenue	revenue = ln size	size represents the principal income balance of a company listed on the stock exchange under the income statement	Cathay Pacific database and author calculations
The pace of primary revenue growth for publicly traded companies	Sale growth	Sale growth = MIC-MIP	MIC represents the main business income of listed companies in the current year; MIP represents the main business income at the beginning of the previous year	Cathay Pacific database and author calculations
The percentage of listed companies' managerial salaries	Salary ratio	Salary ratio = STE-TCP	STE represents the sum of the top three executive team salaries; TCP represents the total compensation paid	Cathay Pacific database and author calculations
How long a listed company has been in business	Firm age	Firm age = OC	OC represents the Operating hours of listed companies	Cathay Pacific database
Listed companies' equity return	roe	roe = NI/NAC	NI represents the Net income; NAC represents the net assets of publicly traded companies	Cathay Pacific database and author calculations
Cost growth rate of listed companies	Cost growth	Cost growth = MEC-MEP	MEC represents the main business cost expenditure of the listed company in the current year; MEP represents the main business cost expenditure at the beginning of the previous year	Cathay Pacific database and author calculations
Listed companies' price-to-earnings ratio	pe	pe = SP/NAC	SP represents the stock price; NAC represents the net assets of publicly traded companies	Cathay Pacific database and author calculations
The properties of listed companies	soe	$${\text{soe = }}\left\{ {\begin{array}{*{20}c} {1} \\ {0} \\ \end{array} } \right.$$	If the listed company is a state-owned enterprise, the value is 1, and the value of non-state-owned enterprise is 0	Cathay Pacific database

#### Data collection

This study did not use any human samples or their data. Using Chinese companies with A share listing in Shanghai and Shenzhen in the period from 2009 to 2019, the current study investigated the link between industrial policy and M&A decisions in a transnational context. The initial sample underwent several screening procedures to ensure the validity of the results: data from the financial industry sample were first excluded, followed by ST-designated data from listed companies, and finally, data with missing values were discarded. In the final version of the publication, all variables that were continuous were trimmed between 1 and 99% to reduce the impact of outliers on the empirical findings. According to the above method, M&A events in the Thomson Reuters SDC Platinum database were screened, and the selected samples were matched with the financial data of the CSMAR database according to stock codes, and listed companies with a severe missing of important financial data were excluded. Finally, 321 cross-border M&A events were successfully matched as the research samples of this paper. Industrial policy data were collected manually for this study. Cathay Pacific's CSMAR database was used as the source of company-level financial statistics. Table 2 displayed the description of statistics for the main variables. In addition, the industries in which the government work report was implemented were determined according to the "Industry Classification Standards for Listed Companies" revised by the China Securities Regulatory Commission in 2012, including agriculture, forestry, animal husbandry, fisheries, business services, and air transport.

**Table 2:** Major variables' descriptive statistics.

variable	Mean	sd	Min	Max	N
cbma	0.0718	0.2582	0.0000	1.0000	25,600.0000
Policy	0.4598	0.4984	0.0000	1.0000	25,600.0000
Size	22.0887	1.4767	15.4177	30.9524	25,600.0000
Asset debt	0.4634	0.6238	0.0071	61.3354	25,600.0000
Roa	0.0410	0.0864	− 6.7139	2.9330	25,600.0000
Dual	0.7698	0.4209	0.0000	1.0000	24,900.0000
Ln sales	21.3214	1.4515	16.7841	25.1007	25,200.0000
Sale growth	1.0280	95.9842	− 1.0455	14,900.0000	25,600.0000
Salary ratio	0.4527	0.1338	0.0882	1.0000	25,600.0000
Firm age	16.2002	5.8055	3.0000	63.0000	25,600.0000
Roe	0.0612	0.5002	− 45.4824	2644	25,500.0000
Cost growth	0.3296	8166	− 0.9788	576.0718	25,200.0000
Ln pe	4.7194	10.2689	− 1.1979	12.9480	22,900.0000

Table 2 showed descriptive statistics for the main variables. The average value of cbma in Table 2 was low. The possible reasons were as follows: (1) the value of most target companies was seriously overvalued, which easily led to the failure of cross-border mergers and acquisitions. (2) Due to cultural conflicts, cultural differences, cultural incompatibilities, and the loss of key personnel, it also hindered the realization of transnational mergers and acquisitions. (3) Information asymmetry and technical differences in the process of M&A affected the results of cross-border M&A. (4) Due to the long geographical distance, the local market situation of the acquired enterprise was not fully understood, and the acquisition price was too high, which increased the financial burden of the enterprise and had a negative effect on the stock price.

As more and more listed companies engaged in mergers, acquisitions, and restructurings, the role of industrial policy in China's capital markets was crucial. This paper analyzed the relationship between industrial policy and Chinese enterprises' cross-border M&A premium and its theoretical mechanism to provide a theoretical basis for listed companies to realize cross-border M&A. At the same time, more and more listed companies participated in mergers and acquisitions, which further highlighted the importance of industrial policy in China's capital market. This paper examined whether there was a correlation between the success rate of cross-border M&A of Chinese enterprises and industrial policy, and provided some enlightenment for guiding industrial policy to promote enterprises' cross-border M&A decision-making. Although the average value of cbma was low, it was still meaningful to study listed companies.

## Results and discussion

### Baseline analysis

This study used a two-way fixed-effect regression based on module to analyze the impact of industrial policy of Chinese on the M&A decisions in transnational of low-listed Chinese companies. Table 3 showed The Influence of Industrial Policy on International Merger and Acquisition Decision-Making. With column 1 excluding control variables and column 2 adding a control variable, columns 1 and 2 did not have a two-way fixed effect. Columns 3 and 4 included a two-way fixed effect, with column 4 adding a control variable, while columns 3 and 4 did not. The findings demonstrated that whether or not the two-way fixed effect was taken into account, as well as whether or not control variables were included, the coefficient of policy or the coefficient of industrial strategy was considerably positive. These findings implied that industrial policy significantly influenced Chinese listed companies that decided to engage in M&A activity around cross-broad field. Column 5 was the regression result of the LPM model on the impact of industrial policy on international M&A decision-making. The result showed that industrial policy significantly promoted international M&A decision-making at the 1% level, indicating that industrial policy support could not only improve the cooperation intention of external capital and listed companies, that is, the possibility of setting up M&A funds in partnership was higher, but also expanded the cooperation depth of external capital. That is, the proportion of external capital invested in M&A funds was higher. In summary, Hypothesis 1.2 was rejected, while Hypothesis 1.1 was confirmed.

**Table 3:** The influence of industrial policy on international merger and acquisition decision− making.

	(1) cbma	(2) cbma	(3) cbma	(4) cbma	(5) cbma
Policy	0.030*** (9.232)	0.031*** (7.823)	0.021** (2.362)	0.027** (3.241)	0.044*** (6.924)
Size		0.018*** (3.971)		0.032*** (3.425)	0.021*** (3.631)
Asset debt		− 0.028 (− 1.628)		0.089*** (0.65)	− 0.013 (− 1.24)
Roa		0.267*** (1.32)		0.370*** (2.47)	0.124*** (1.013)
Dual		− 0.018*** (− 4.871)		− 0.020*** (− 2.981)	− 0.011*** (− 2.971)
Lnsales		− 0.004 (− 1.124)		− 0.016*** (− 2.823)	− 0.002 (− 0.924)
Sale growth		− 0.001** (− 2.427)		− 0.001* (− 1.917)	− 0.002* (− 1.351)
Salary ratio		− 0.041*** (− 1.92)		0.009 (0.413)	− 0.021*** (− 1.381)
Firm age		− 0.002*** (− 3.342)		0.018 (0.991)	− 0.001*** (− 2.342)
Roe		− 0.062** (− 2.091)		− 0.062 (− 1.241)	− 0.043*** (− 1.902)
Cost growth		0.002*** (3.241)		0.002** (2.421)	0.001*** (0.193)
Ln pe		0.002 (0.031)		− 0.002 (− 0.026)	0.001 (0.014)
Effect of individual fixation	No	No	Yes	Yes	Yes
The Effect of time fixation	No	No	Yes	Yes	Yes
_Cons	0.058*** (26.533)	− 0.021 (− 1.001)	0.062*** (1395)	− 0.214 (− 1.093)	− 0.041 (− 2.724)
R^2^	0.003	0.008	0.233	0.253	0.106
N	25,645	21,756	25,208	21,272	21,272

z statistics in brackets.

*p < 0.1, **p < 0.05, ***p < 0.01.

### Impact mechanism test

#### Test of the financial constraint mechanism (FCM)

This study employed the stepwise regression approach based on Eqs. (1) and (3) to examine how industrial policy might have encouraged cross-border M&A by easing financial restrictions on businesses. Table 4 showed the Capacity for securing financing for businesses. Long-term assets, which indicated the financing capacity of a company, were the explanatory variable in columns 1 through 4, whereas industrial policy was the explanatory variable in columns 2 and 4. Control variables were present in columns 2 and 4, but not in columns 1 and 3. The findings showed that even after controlling for two-way fixed effects as well as control variables, the coefficient of Chinese industrial policy was still considerably favorable, suggesting that industrial policy increased companies' financing capacity and alleviated their financial constraints. Columns 5 and 6 used cbma, or whether or not a company made an acquisition in the cross-border field, as the explanatory variable. After accounting for two-way fixed effects as well as control variables, the coefficients of Chinese Industrial Politics and long assets were significantly positive, satisfying the requirements of the intermediation effect model, arguing that industrial policy might have used a company's finance capabilities as a means of mediation to encourage cross-border M&A. Therefore, Hypothesis 2.1 was validated.

**Table 4:** Capacity for securing financing for businesses.

	(1) long_asset	(2) long_asset	(3) long_asset	(4) long_asset	(5) cmba	(6) cmba
Policy	0.009*** (7.525)	0.002* (1.824)	0.007*** (3.383)	0.012*** (2.418)		0.023* (1.918)
long_asset					0.027*** (2.752)	0.012*** (2.752)
Size		0.061*** (59.942)		0.041*** (24.314)	0.028*** (3.874)	0.027*** (3.862)
Assetdebt		0.176*** (51.418)		0.165*** (39.424)	0.032** (2.214)	0.031** (2.341)
Roa		0.076*** (0.613)		0.024* (1.652)	0.283*** (2.134)	0.298*** (2.124)
Dual		0.012*** (8.413)		0.002 (1.452)	− 0.009** (− 1.532)	− 0.008** (− 1.982)
Lnsales		− 0.024*** (− 50.134)		− 0.034*** (− 17.422)	− 0.009** (− 2.014)	− 0.009** (− 2.017)
Sale growth		0.000 (0.524)		0.000 (1.001)	− 0.000* (− 1.632)	− 0.000* (− 1.324)
Salary ratio		0.023** (2.641)		0.003 (0.712)	0.018 (0.654)	0.018 (0.639)
Firm age		− 0.001*** (− 5.542)		− 0.009*** (− 3.752)	0.021 (1.313)	0.019 (0.989)
Roe		− 0.021** (− 1.991)		− 0.031*** (− 3.314	− 0.032 (− 0.872)	− 0.032 (− 0.814)
Cost growth		− 0.000 (− 0.635)		− 0.000 (− 1.231)	0.001** (2.313)	0.001** (2.431)
Ln pe		− 0.001 (− 0.452)		0.001 (0.092)	− 0.001 (− 0.081)	− 0.001 (− 0.063)
Individual fixation effect	No	No	Yes	Yes	Yes	Yes
Time fixation effect	No	No	Yes	Yes	Yes	Yes
_Cons	0.061*** (75.716)	− 0.323*** (− 22.091)	0.067*** (58.572)	− 0.099* (− 1.642)	− 0.582* (− 1.872)	− 0.525* (− 1.535)
R^2^	0.003	0.353	0.642	0.772	0.342	0.282
N	21,983	18,687	21,703	18,370	18,370	18,370

z data enclosed in brackets.

*p < 0.1, **p < 0.05, ***p < 0.01.

#### The mechanism of industry political relevance

Equation (4) was used in this work to conduct an empirical investigation into the process by which companies' political relevance affected the effect of Chinese industrial policy on their choice to undertake transnational mergers and acquisitions. Table 5 showed the empirical results of the mechanism of political relevance. Two-way fixed effects were included in columns 3 and 4, but not in columns 1 and 2 above. Control variables were included in columns 2 and 4, but not in columns 1 and 3 above. By using two-way fixed effects or by controlling for variables, the results showed that the coefficient on the Chinese industrial policy variable was quite positive. These results suggested that, with the help of industrial policy, soes were increasingly assuming political responsibility and thus becoming the driving force behind international mergers and acquisitions. Thus, Hypothesis 2.2 is confirmed.

**Table 5:** Mechanism for political relevance.

	(1) cmba	(2) cmba	(3) cmba	(4) cmba
Policy	0.032*** (7.689)	0.028*** (5.952)	0.025** (2.139)	0.033** (2.342)
Soe	− 0.018*** (− 1.452)	− 0.023*** (− 0.352)		
c.policy#c.soe	0.011*** (2.625)	0.012*** (2.314)	0.018*** (3.573)	0.023*** (3.525)
Size		0.011*** (1.358)		0.018*** (3.135)
Assetdebt		− 0.013 (− 0.883)		0.074*** (0.358)
Roa		0.219*** (3.752)		0.263*** (2.553)
Dual		− 0.009*** (− 3.242)		− 0.014*** (− 2.352)
Lnsales		− 0.002 (− 0.872)		− 0.015*** (− 2.642)
Sale growth		− 0.000** (− 2.324)		− 0.000* (− 1.642)
Salary ratio		− 0.0243*** (− 0.824)		0.009 (0.642)
Firm age		− 0.001*** (− 3.153)		0.012 (0.835)
Roe		− 0.084** (− 2.524)		− 0.032 (− 1.342)
Cost growth		0.002*** (2.752)		0.001** (2.322)
Ln pe		− 0.000 (− 0.073)		− 0.000 (− 0.013)
Individual fixation effect	No	No	Yes	Yes
Time fixation effect	No	No	Yes	Yes
_Cons	0.066*** (22.391)	− 0.082** (− 2.145)	0.061*** (1145)	− 0.424 (− 1.244)
R^2^	0.005	0.019	0.233	0.257
N	25,644	21,756	25,207	21,272

z statistics in parentheses.

*p < 0.1, **p < 0.05, ***p < 0.01.

Table 6 showed the use of "biographies of Board members" as an instrumental variable to measure "political relevance". The regression results showed that the regression results were basically consistent with those in Table 5, and were significantly positive at the 1% significance level. Then, with the help of industrial policies, state-owned enterprises increasingly assumed political responsibilities to promote international mergers and acquisitions. Therefore, the results obtained in this paper were robust.

**Table 6:** Instrumental variable method regression results.

	(1) cmba	(2) cmba	(3) cmba	(4) cmba
Policy	0.042** (2.317)	0.035** (2.041)	0.046* (1.082)	0.039* (1.720)
Rbm	− 0.024*** (− 1.342)	− 0.014*** (− 0.422)		
c.policy#c.rbm	0.032*** (3.542)	0.028*** (2.135)	0.032*** (4.672)	0.016*** (0.231)
Size		0.032*** (1.021)		0.019*** (3.231)
Assetdebt		− 0.009 (− 0.141)		0.052*** (0.321)
Roa		0.140*** (3.024)		0.134*** (0.531)
Dual		− 0.010*** (− 2.352)		− 0.021*** (− 1.982)
Lnsales		− 0.001 (− 0.321)		− 0.031*** (− 3.341)
Sale growth		− 0.000** (− 1.241)		− 0.000* (− 1.023)
Salary ratio		− 0.021*** (− 0.241)		0.031 (0.782)
Firm age		− 0.001*** (− 2.314)		0.012 (0.314)
Roe		− 0.0231** (− 2.134)		− 0.031 (− 1.092)
Cost growth		0.002*** (2.213)		0.001** (2.013)
Ln pe		− 0.000 (− 0.002)		− 0.000 (− 0.002)
Individual fixation effect	No	No	Yes	Yes
Time fixation effect	No	No	Yes	Yes
_Cons	0.009*** (19.532)	− 0.032** (− 1.131)	0.082*** (1.344)	− 0.147 (− 0.831)
R^2^	0.002	0.027	0.325	0.314
N	25,644	21,756	25,207	21,272

### Robustness analysis

For the purpose of increasing the robustness of the results and mitigating potential sample selection bias and endogeneity issues, this study used propensity score matching to reselect the sample and conduct further robustness analysis. A statistical technique called propensity score matching was used to lessen bias and confounding factors in observational research, enabling a more fair comparison of experimental and control groups. Because there might have been non-random selection factors in industrial policies, which might have resulted in certain endogenous problems, this paper further used the propensity score matching method (PSM) for testing. Propensity score matching helped to improve the problem of sample selection bias and ensure the comparability of the treatment group and control group. The specific operation was as follows: first, the control variable was used as a covariate to match the propensity score year by year, and then the samples were matched with 1:1 proximity to obtain the successfully matched samples. The regression test was conducted using the samples matched by propensity score. The regression results were shown in column (5) of Table 7, and the main conclusions did not change.

**Table 7:** Robustness test: independent variable lag one stage, propensity score matching and dynamic panel model.

	(1) cmba	(2) cmba	(3) cmba	(4) cmba	(5) cbma	(6) cbma
Policy					0.026*** (3.039)	
${\mathrm{pcbmapre}}_{\mathrm{it}-1}$						0.307*** (4.023)
Lpolicy	0.028*** (9.134)	0.029*** (6.124)	0.021** (1.928)	0.027** (2.123)		0.031*** (3.921)
Size		0.013*** (2.873)		0.023*** (2.134)	0.014 *** (5.571)	0.072*** (3.923)
Assetdebt		− 0.002 (− 0.104)		0.102*** (3.231)	0.082** (2.184)	0.001** (1.982)
Roa		0.321*** (5.134)		0.431*** (4.231)	0.135** (2.091)	0.391** (2.125)
Dual		− 0.0752*** (− 9.024)		− 0.031*** (− 3.023)	− 0.013*** (− 3.013)	− 1.452 (− 0.034)
Lnsales		− 0.004* (− 1.024)		− 0.021** (− 2.013)	− 0.002** (− 2.341)	− 0.241* (− 2.019)
Sale growth		− 0.001 (− 0.231)		0.012 (0.112)	0.002 (0.023)	0.132 (0.120)
Salary ratio		0.021** (1.972)		0.023 (1.032)	0.026 (1.024)	0.092 (1.291)
Firm age		− 0.000 (− 0.723)		0.012 (1.115)	0.091 (1.223)	0.033 (1.345)
Roe		− 0.092*** (− 2.024)		− 0.102 (− 1.092)	− 0.013 (− 0.913)	− 0.034 (− 0.871)
Cost growth		0.002 (1.001)		0.001 (0.023)	0.001 (0.021)	0.004 (0.425)
Ln pe		0.000** (2.103)		0.000** (2.023)	0.001** (2.391)	0.007*** (2.814)
Individual fixation effect	No	No	Yes	Yes	Yes	Yes
Time fixation effect	No	No	Yes	Yes	Yes	Yes
_Cons	0.078*** (2690)	0.013 (0.431)	0.085*** (13.752)	− 0.231 (− 1.321)	− 0.291 (− 1.102)	− 0.832 (− 1.013)
R^2^	0.005	0.013	0.215	0.212	0.291	0.313
N	14,523	10,924	14,345	10,352	12,782	12,782
P-AR(1)						0.040
P-AR(2)						0.651

z data enclosed in brackets.

*p < 0.1, **p < 0.05, ***p < 0.01.

In order to avoid possible reverse causality, this paper carried out regression for independent variables with a lag of one cycle. The results were shown in the first, second, third, and fourth columns of Table 7. The regression results were consistent with the benchmark regression results in Table 3, and were all significantly positive at the 1% level, so the robustness of the regression results could be tested.

Column 6 of Table 7 indicated that the dynamic panel model was used for a robustness test, and the lag term of the explained variable was added to the model for dynamic panel analysis. The results showed that the P-values of the first-order autocorrelation test results were mostly less than 0.05, and the P-values of the second-order autocorrelation test results were all greater than 0.1, indicating that the first-order difference of the disturbance term had autocorrelation, while the second-order difference had no autocorrelation. The hypothesis that the perturbation term had no autocorrelation was satisfied. It showed that the model setting was valid.

Tables 8 and 9 provided, respectively, the regression findings for the baseline adjustment and the annual adjustment. The findings demonstrated that the coefficient of Chinese industrial policy or industrial policy was still considerably positive even when two-way fixed effects and control variables were taken into consideration. This supported the findings of this study and further ruled out endogeneity problems by showing that industrial policy had a considerable favorable influence on cross-border M&A decisions around cross-border field.

**Table 8:** Results of regression after adjustment of the baseline.

	(1) cmba	(2) cmba	(3) cmba	(4) cmba
Policy	0.044*** (9.392)	0.036*** (6.982)	0.025** (2.009)	0.029** (2.224)
Size		0.012*** (2.742)		0.027*** (2.562)
Assetdebt		− 0.002 (− 0.122)		0.112*** (3.536)
Roa		0.424*** (5.324)		0.532*** (1.353)
Dual		− 0.042*** (− 10.232)		− 0.021*** (− 2.241)
Lnsales		− 0.005* (− 1.762)		− 0.024** (− 2.321)
Sale growth		− 0.001 (− 0.424)		0.013 (0.134)
Salary ratio		0.032** (2.124)		0.021 (1.042)
Firm age		− 0.000 (− 0.813)		0.021 (1.121)
Roe		− 0.142*** (− 3.143)		− 0.113 (− 1.313)
Cost growth		0.002 (1.013)		0.001 (0.212)
Ln pe		0.000** (2.824)		0.000** (2.241)
Individual fixation effect	No	No	Yes	Yes
Time fixation effect	No	No	Yes	Yes
_Cons	0.078*** (2690)	0.042 (0.742)	0.085*** (13.752)	− 0.681 (− 1.542)
R^2^	0.005	0.024	0.215	0.241
N	16,577	14,353	16,427	14,180

z data enclosed in brackets.

*p < 0.1, **p < 0.05, ***p < 0.01.

**Table 9:** Results of regression after annual adjustment.

	(1) cmba	(2) cmba	(3) cmba	(4) cmba
Policy	0.030* (1.802)	0.045*** (3.673)	0.088* (1.785)	0.014** (2.341)
Size		0.062*** (6.022)		0.076*** (2.532)
Assetdebt		0.163*** (2.135)		0.253*** (2.421)
Roa		− 2.134*** (− 1.141)		− 1.532*** (− 0.325)
Dual		0.331*** (36.242)		0.241*** (10.642)
Lnsales		− 0.042*** (− 5.013)		− 0.042* (− 1.763)
Sale growth		− 0.000 (− 1.541)		− 0.002 (− 0.435)
Salary ratio		1.441*** (26.652)		1.221*** (9.752)
Firm age		− 0.004*** (− 3.562)		0.324 (0.724)
Roe		− 0.143*** (− 2.135)		− 0.135 (− 1.234)
Cost growth		0.001 (1.034)		0.003 (0.424)
Ln pe		0.000 (0.103)		− 0.000 (− 0.152)
Effect of individual fixation	No	No	Yes	Yes
Time fixation effect	No	No	Yes	Yes
_Cons	0.483*** (39.068)	− 0.463*** (− 3.145)	0.403*** (1514)	− 2.454 (− 1.432)
R^y^	0.001	0.531	0.717	0.762
N	3628	3425	2818	2639

z data enclosed in brackets.

*p < 0.1, **p < 0.05, ***p < 0.01.

### Heterogeneity analysis of cross-border mergers and acquisitions in the same industry and non-same industry

Based on the study of Yang^49^, this paper classified the original sample data from the industry level into the event level. The regression results showed that industrial policy had a significant positive promoting effect on both peer and non-peer cross-border mergers and acquisitions. Among them, the promotion effect of industrial policy on cross-border mergers and acquisitions of the same industry was slightly more significant than that of industrial policy on cross-border mergers and acquisitions of non-peers. Table 10 showed the heterogeneity analysis results of cross-border mergers and acquisitions in the same industry and non-same industry.

**Table 10:** Heterogeneity analysis results of cross-border mergers and acquisitions in the same industry and non-same industry.

	cbma	
	Cross-border mergers and acquisitions in the same industry	Cross-border mergers and acquisitions in different industries
Policy	0.032*** (3.782)	0.018*** (2.093)
Control variable	Yes	Yes
Effect of individual fixation	Yes	Yes
The effect of time fixation	Yes	Yes
N	12,821	8451

### Discussion

#### Salutary influence of industrial policies

The results demonstrate that the regression coefficients between industrial policies and the cross-border M&A decisions of listed companies are consistently positive and significant (as shown in Table 3). This suggests that industrial policies have a pronounced impact on cross-sector M&A decisions of Chinese listed firms. Grounded in the Resource-Based View, when an industrial policy supports a particular sector or technology of a listed company, firms within that sector might show heightened investment enthusiasm towards their own resources. This industrial policy, in turn, influences a company's pursuit of its unique resources, subsequently impacting its cross-border M&A decisions^50^. Relying on Transaction Cost Theory, industrial policies, by offering incentives such as loan waivers and tax benefits, effectively reduce the transaction costs associated with cross-border M&As. Therefore, when making decisions, listed companies prioritize transaction costs and returns^51^. According to Market Power Theory, when an industrial policy endorses a specific sector or technology, listed companies, aiming to solidify their market position within that domain, resort to M&As to achieve a larger market share, thereby neutralizing threats from rivals. These theoretical frameworks provide robust support for the findings of this research^52^. From a directional guidance perspective, industrial policies offer clear developmental trajectories for listed companies, aligning with the nation's long-term development goals and M&A strategies. By securing government support, they also foster the accumulation of social capital, thereby further facilitating the implementation of cross-border M&A decisions^53^. From a resource-sharing standpoint, listed companies extract invaluable information about target markets—such as macroeconomic data, regulatory landscapes, and cultural contexts—from industrial policies, providing valuable insights for their cross-border M&A ventures^54,55^. From a technological vantage, industrial policies cater to the technological and knowledge requisites of listed companies, setting the foundation for the technology and knowledge indispensable for their cross-border M&As^56^. These findings articulate the positive role of industrial policies in shaping M&A decisions of listed firms from perspectives of directional guidance, resource-sharing, and technological support. Specifically, industrial policies provide explicit developmental goals for listed entities, bolster market resource-sharing, and encourage their technological innovation prowess.

Thus, industrial policies are instrumental for listed firms in acquiring cutting-edge technologies, managerial expertise, and market resources, ultimately enhancing their global market competitiveness, which holds profound significance in propelling national technological innovation, resource optimization, and economic growth.

#### Constructive effects of financing constraints

The findings of this research indicate that industrial policies enhance the financing capacity of enterprises (as shown in Table 4), alleviate their financing constraints, and foster cross-border M&A decisions for listed companies. Industrial policies can mitigate corporate financing constraints through policy orientation^30,47^, credit constraints^57^, and guiding capital flows^58^. As these financing constraints are relaxed and financing capability bolstered, risks associated with cross-border M&As decrease, granting firms a more flexible array of M&A structural options, consequently promoting the occurrence of cross-border M&As. While the majority of studies on the impact of industrial policies on M&A decisions of listed companies mainly focus on how such policies influence market structures^59^, technological innovation^60^, and corporate investments^61^, there is limited research directly examining their effect on corporate financing capacity. Moreover, the bulk of existing literature predominantly studies the influence of economic environments^62^, financial markets^63^, and corporate governance on financing constraints. This study reveals the operational mechanism and characteristics of industrial policies impacting listed companies' cross-border M&As, highlighting the role of financing constraints. It is found that industrial policies effectively boost firms' financing capacity by reducing financing costs, expanding financing channels, and optimizing financing structures, thus furnishing greater opportunities and resources for cross-border M&As. On one hand, industrial policies, via tax breaks, low-interest loans, and fiscal subsidies, can offer businesses a low-cost source of funds, thereby diminishing financing barriers^39^. On the other hand, industrial policies support financial innovation, liberalize financial market entries, and improve financial conditions, affording SMEs various financing avenues. Additionally, industrial policies can incentivize firms to refine their financing structures, reducing liquidity risks. A company's financing capacity is a crucial determinant influencing its cross-border M&A decision-making and execution. Ample financial strength positions firms advantageously during M&A negotiations and ensures a smoother integration post-acquisition^64^. Therefore, through various means, industrial policies promote corporate financing capacity, subsequently providing robust support for businesses' cross-border M&As.

#### Pivotal influence of political relevance

Regression results indicate that industrial policies can promote cross-border M&As by enhancing political affiliations (as shown in Table 5). While existing studies delve into the themes of industrial policies, political ties, and cross-border M&As, the relationship among these three remains a relatively uncharted territory. Firstly, during the implementation of industrial policies, governments often exhibit control over certain resource allocations. Consequently, firms are inclined to establish close ties with organizations controlling these resources, making political affiliations the most prevalent intimate relationship formed between businesses and governments. The political ties that firms foster also assist them in obtaining timely and accurate information regarding governmental innovation strategies and industrial policies^65^. Secondly, in sectors where state-owned enterprises (SOEs) dominate or where government participation is significant, governments often establish close political ties with firms, either through equity holdings or other means. SOEs typically possess stronger financial capabilities and can secure funds at lower borrowing costs, giving them a clear financial edge in cross-border M&A activities. They can raise funds required for M&As at reduced costs^66^. Additionally, the relative stability and lower financial risks of SOEs provide them with broader strategic choices when conducting cross-border M&As. They can undertake long-term investments and expansions while minimizing risks posed by short-term financial turbulences^67^. Furthermore, this study employs the “biographies of Board members” as an instrumental variable to measure "political relevance". Board members play pivotal roles in decision-making processes (as shown in Table 6). When it comes to long-term strategies and major investments, their extensive experience, knowledge repository, and robust relational networks facilitate the identification, evaluation, and execution of M&A opportunities. Industrial policies offer targeted support for board members in training and education, bolstering firms' global market competitiveness and M&A capabilities. On the one hand, industrial policies encourage board members to participate in international conferences and seminars, fostering international networking and laying a robust foundation for cross-border M&As^68^. On the other hand, board decisions hinge on M&A targets and clear strategic directions provided by industrial policies^69^. Therefore, by influencing the selection, training, and networking of board members, industrial policies can indirectly shape a company's cross-border M&A strategy.

#### Limitations and future research

Firstly, China's industrial policies are rich and diverse, with different industries and regions potentially exhibiting distinct policy features and implementation intensities. This study may not have delved deeply into these nuances, potentially leading to some generalizations. Given the richness and diversity of China's industrial policies, future research can explore more deeply the relationship between policies and cross-border M&A activities in specific industries or sub-industries. For instance, high-tech, manufacturing, and service sectors can be separately investigated, analyzing their cross-border M&A dynamics and characteristics under policy support. Future studies could also further examine the relationship between the enforcement strength of industrial policies in different Chinese regions and firms' cross-border M&A activities. For example, comparing coastal eastern regions with inland western areas, one can analyze how local governments differentiate in the enforcement of industrial policies within the same national policy framework, and how these variances impact local firms' cross-border M&A decisions.

Secondly, when studying cross-border M&A decisions, an overseas M&A event must be announced to finalize the acquisition. In comparison, the success rate of cross-border M&As in our country is not particularly high, which could influence the results. Future research can pay more attention to and delve into unannounced cross-border M&A activities. For example, researchers can collect relevant information about non-publicized M&A events through interviews, surveys, or data sharing with partners. Additionally, one can explore why certain companies choose not to announce their M&A activities, and the strategic intentions and business considerations behind such decisions. Regarding the not-so-high success rate of cross-border M&As in our country, future studies can specifically explore the exact reasons for failures. For instance, comparative research between our country and other nations on M&A success rates could unearth potential differences related to geographical, industrial, cultural, and managerial factors.

## Conclusions

For the purpose of assess the influence of Chinese industrial policy on Chinese companies’ M&A decisions around cross-border context, while taking into consideration the production environment of companies, this study employed a two-way fixed-effects model and and a mechanism of influence effect model. The approach applied was that of propensity score matching in the study to eliminate the bias caused by sample selection and examine two mechanisms of Chinese industrial policy to promote M&A in the field of transnational trade. The following are the study's primary conclusions:

1. Industrial policy uses financial constraints and policy correlation mechanisms to influence cross-border M&A.
2. By easing financial restrictions, industrial policy may improve company ability to obtain money and encourage cross-border M&A.
3. State-owned enterprises have high political relevance, and their decisions are critical to industrial policies that support international mergers and acquisitions.

Overall, this research offers insights into the mechanisms of Chinese industrial policy that promote M&A in cross-border and emphasizes the importance of considering the production environment and political relevance of firms. The results of this study have significant ramifications for corporations looking to conduct cross-border M&A activity as well as governments.

## Theoretical and practical implications

### Theoretical implications

This study offered a fresh perspective in the field of industrial policy, bearing significant theoretical implications, as elaborated below:

1. Clarifying the relationship between industrial policies and cross-border M&As. Through an in-depth analysis of data from 2009 to 2019, this paper enriches and refines the existing theoretical framework regarding the relationship between industrial policies and cross-border M&As. This study not only reveals the direct relationship between the two but also delves into the potential underlying mechanisms, offering a more systematic and detailed understanding for the academic community.
2. Deepening the understanding of the influence of political factors on economic decisions. This research examines the role of high political connectedness in state-owned enterprises in cross-border M&As, an area not yet sufficiently addressed in the current literature. This finding reinforces the theoretical understanding of the interaction between politics and economics, especially in the context of China's political-economic landscape.
3. Enriching the theory of risk and reward assessment in cross-border M&As. Through this study, we have gained a clearer understanding of the impact of industrial policies on the risks and rewards of cross-border M&As. This offers theoretical backing for businesses in their practical operations, assisting them in making more informed decisions.

### Practical implications

This research held significant practical value, offered actionable insights and guidance for industrial decision-makers and policymakers in this field, as detailed below:

1. Offering insights for policy formulation. The findings of this study reveal the positive influence of industrial policies on corporate cross-border M&A decisions, especially in enhancing firms' capital acquisition and amplifying the political connectedness of state-owned enterprises. By investigating the relationship between industrial policies and cross-border M&As, governments can more precisely understand which policy elements are most effective in promoting firms' overseas acquisition activities. Authorities and relevant decision-making bodies can draw on these results, recognizing that given China's vast regions and diverse industries, different areas and sectors might require distinct industrial policies to drive their cross-border M&A endeavors. Based on the insights from this research, governments can more accurately devise or adjust industrial policies for various regions and industries, maximizing the benefits of cross-border M&As.
2. Propelling financial reforms. The research suggests that relaxing financial restrictions can empower firms to enhance capital acquisition, thereby encouraging them to undertake cross-border M&As. This underscores the urgency of financial system reforms, especially in providing businesses with more flexible and efficient financing avenues. The study indicates that industrial policies can heighten the political connectedness of state-owned enterprises, subsequently boosting the success of their cross-border M&A endeavors. This implies that during financial reforms, governments and financial institutions should prioritize collaboration with state-owned enterprises, ensuring that financial resources effectively back their overseas acquisition activities.
3. Offering theoretical support for M&A risk management. When companies embark on cross-border M&As, they must rationally evaluate the risks and rewards involved. This research advises firms to consider not just the economic benefits during cross-border M&A decisions but also to carry out comprehensive risk assessments aligned with industrial policies. This aids businesses in ensuring M&A success while minimizing uncertainties and risks in the process. The findings from this paper provide crucial theoretical guidance for firms, helping them manage risks more systematically, ensuring the successful execution of M&A activities.

## Data Availability

The datasets used and/or analysed during the current study available from the corresponding author on reasonable request.
